# Modelling the Influence of Electromagnetic Field on the User of a Wearable IoT Device Used in a WSN for Monitoring and Reducing Hazards in the Work Environment

**DOI:** 10.3390/s20247131

**Published:** 2020-12-12

**Authors:** Patryk Zradziński, Jolanta Karpowicz, Krzysztof Gryz, Leszek Morzyński, Rafał Młyński, Adam Swidziński, Konrad Godziszewski, Victoria Ramos

**Affiliations:** 1 Laboratory of Electromagnetic Hazards, Central Institute for Labour Protection—National Research Institute (CIOP-PIB), Czerniakowska 16, 00-701 Warszawa, Poland; jokar@ciop.pl (J.K.); krgry@ciop.pl (K.G.); 2 Department of Vibroacoustic Hazards, Central Institute for Labour Protection—National Research Institute (CIOP-PIB), Czerniakowska 16, 00-701 Warszawa, Poland; lmorzyns@ciop.pl (L.M.); rmlynski@ciop.pl (R.M.); adswi@ciop.pl (A.S.); 3 Institute of Radioelectronics and Multimedia Technology, Warsaw University of Technology, Nowowiejska 15/19, 00-665 Warszawa, Poland; K.Godziszewski@ire.pw.edu.pl; 4 Telemedicine and e-Health Research Unit, Instituto de Salud Carlos III, Avda. Monforte de Lemos, 5, 28029 Madrid, Spain; vramos@isciii.es

**Keywords:** biomedical engineering, environmental engineering, Internet of Things (IoT), numerical simulations, radiofrequency sensor, occupational exposure, public health, specific energy absorption rate (SAR), wearables, wireless sensor network (WSN)

## Abstract

The aim of this study was to evaluate the absorption in a user’s head of an electromagnetic field (EMF) emitted by the Wi-Fi and/or Bluetooth module of a wearable small Internet of Things (IoT) electronic device (emitting EMF of up to 100 mW), in order to test the hypothesis that EMF has an insignificant influence on humans, and to compare the levels of such EMF absorption in various scenarios when using this device. The modelled EMF source was a meandered inverted-F antenna (MIFA)-type antenna of the ESP32-WROOM-32 radio module used in wearable devices developed within the reported study. To quantify the EMF absorption, the specific energy absorption rate (SAR) values were calculated in a multi-layer ellipsoidal model of the human head (involving skin, fat, skull bones and brain layers). The obtained results show up to 10 times higher values of SAR from the MIFA located in the headband, in comparison to its location on the helmet. Only wearable IoT devices (similar in construction and way of use to the investigated device) emitting at below 3 mW equivalent isotropically radiated power (EIRP) from Wi-Fi/Bluetooth communications modules may be considered environmentally insignificant EMF sources.

## 1. Introduction

### 1.1. Wireless Sensor Network (WSN) and Internet of Things (IoT) Technologies

In Internet of Things (IoT) systems, each device has the ability to collect and then transfer data between devices, via wired or wireless networks, using an electromagnetic field (EMF) from the radiofrequency band. Depending on the required range and data transfer rate of wireless connectivity, a number of technologies may be applied: Wireless Fidelity (Wi-Fi), Bluetooth, ZigBee, Long Range (LoRa), RadioFrequency IDentification (RFID) and four to five generations of mobile communication systems (4G–5G), as well as Long Term Evolution (LTE) [1].

A major technology enabling IoT is the Wireless Sensor Network (WSN). A WSN can be defined as a group of spatially deployed sensors and actuators (recognised as nodes) used to monitor or control physical systems or environmental conditions. Each node (from a few to a few thousand) of the WSN is connected to one or more nodes. The nodes communicate with each other using radio signals.

WSNs can be used for various purposes, such as intelligent transportation systems and vehicular communications; electricity, gas and water monitoring; and smart homes, cities and factories (Industry 4.0) [1,2,3,4]. They are also used to monitor various bodily functions, typically in e-Health solutions but also for firefighters, industry and military workers, to detect firefighter movements or track soldiers and predict the warzone [5,6,7,8,9,10,11,12,13,14].

The WSN and IoT can also be used to monitor the level of harmful agents in the work environment (such as noise), or to reduce hazards to workers’ health and safety related to environmental agents [15]. In such applications, the protected person often has to be equipped with a wearable device, either a sensor or an actuator, which is necessary to assess the monitored parameters of exposure or evaluate the hazards, or is aimed at reducing them. Such a sensor or actuator (usually a small wireless device in various shapes and forms) can be worn, for example, in a way comparable to a watch or some kind of band, or can be built-in or attached to various items of work clothes or personal protective equipment (such as a helmet, life vest or mask). In the further part of the article, two examples of WSNs developed at the Central Institute for Labour Protection—National Research Institute (CIOP-PIB), Poland, aiming at monitoring and reducing hazards in the work environment are presented. They include wearable devices whose construction is based on the ESP32-WROOM-32 radio communication module.

### 1.2. Examples of Wearable WSN and IoT Devices to Monitor and Reduce Hazards in the Work Environment

**Figure 1:**
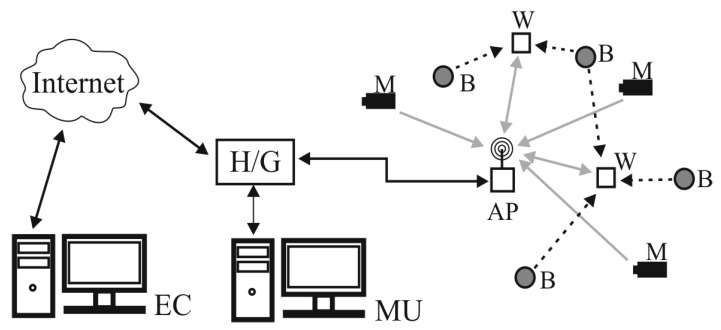
Basic structure of a wireless sensor network for monitoring the work environment and alerting workers to hazards (M—measuring device; W—wearable device; B—Bluetooth beacon; AP—Wi-Fi access point; MU—network main unit; H/G—network hub and gate; EC—external computer; grey arrows—Wi-Fi connections; black dashed arrows—Bluetooth connections; black arrows—Ethernet connections).

WSNs and the IoT can be used for various purposes in the work environment. WSNs used for monitoring the work environment and alerting workers to hazards consist of measuring devices, wearable devices and Bluetooth beacons (Figure 1) [15,16]. Measuring (sensor) devices are deployed in the workplace area and used to monitor harmful agents in specific areas of the workplace. Sensor devices using Wi-Fi connections send the data to the network main unit (MU) to processes them in order to assess hazards in the workplace area. The collected data can also be analysed using an external computer (EC) connected to the Internet. Wearable devices are used to alert workers to hazards and inform them of the necessary safety measures. They communicate with the network and MU using Wi-Fi, and are also used to determine whether the worker should be alerted when approaching the area under any hazard (i.e., danger zone). The location of the worker is evaluated based on the level of electromagnetic signal received by wearable devices from Bluetooth beacons deployed in the workplace.

**Figure 2:**
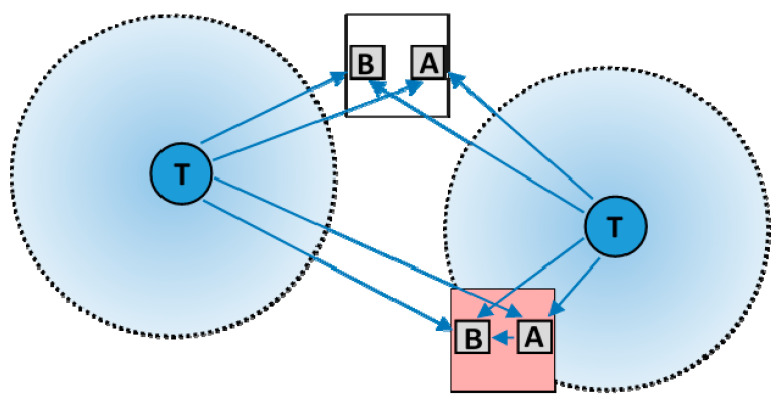
Plan view of basic structure of a system warning against approaching vehicles for workers using hearing protectors in the noisy work environment (T—transmitter mounted on a moving vehicle; A and B—pairs of wearable basic (**B**) receivers and additional (**A**) receivers/transmitters; blue arrows—Bluetooth connections; dashed circle—danger zone boundary defined by the selected received signal strength indicator (RSSI) value).

Another example is a system warning against approaching vehicles, dedicated for workers using hearing protectors for reducing direct acoustic hazards in the work environment [17]. It consists of three types of component: transmitters mounted on every vehicle moving around the workplace, and wearable—basic (primary) and additional (secondary) receivers—used by each worker having access there, as shown in Figure 2. The operation of the WSN is based on an analysis of the strength of the received signal, which is a metric of the distance between the receiver (worker) and the transmitter (vehicle). It is implemented with the use of a received signal strength indicator (RSSI) that can be read by a microprocessor. Transmitters mounted on every vehicle are Bluetooth beacons broadcasting specific radio signals with a constant transmission power. The danger zone around the vehicle is defined as a minimum distance from a vehicle, within which the worker should be warned about a moving vehicle. This distance, at the constant power of the transmitter, also sets out the minimum strength of the signal received by wearable devices, above which the worker should be warned. In this way, the system reduces the risk that the worker is hit by approaching vehicle.

The wearable basic (primary) signal receiver was designed to be worn on the wrist by a worker and is integrated with alarm systems producing vibration (haptic), light and sound warning signals, of which the vibration signal is best received in a noisy e.g., industrial work environment. The Bluetooth radio signal (at 2.4 GHz frequency) can be highly attenuated in the human body (in the field tests, RSSI can differ by up to 20 dBm depending on the position of the worker in relation to the transmitter). Because of that, an additional (secondary) receiver, attached to the worker’s helmet (on the opposite side of the body to the basic receiver) was used to improve the directional sensitivity of the system. Based on an analysis of the level of the signals received by the basic and additional receivers, the warnings to the worker are either generated or not. For this purpose an additional transmitter/receiver (located at the helmet) communicates with the basic receiver (located at the wrist) using Bluetooth communication technology.

### 1.3. Bluetooth and Wi-Fi Wireless Communication Technologies

Wi-Fi is local wireless network technology using Wireless Local Area Network (WLAN) based on IEEE 802.11 standards. Wi-Fi uses unlicensed industrial, scientific and medical (ISM) frequency bands: (2400–2483.5) MHz—known as Wi-Fi 2G, and (5150–5350) MHz and (5470–5725) MHz—known as Wi-Fi 5G. The recently introduced Wi-Fi HaLow is a Wi-Fi extension that enables the low energy connectivity required for sensor and wearable applications, smart homes, autonomous vehicles and smart cities. Wi-Fi HaLow is based on the 802.11ah standard and operates in the 900 MHz frequency band. The maximum operational range of Wi-Fi is 70 m indoors and 1000 m outdoors. The limits of equivalent isotropically radiated power (EIRP) for Wi-Fi devices used without special administrative permission are (1) 100 mW (20 dBm) for Wi-Fi 2G (European Telecommunications Standards Institute (ETSI) EN 300–328 v2.2.2 (2019-07)); (2) 200 mW (23 dBm) for (5150–5350) MHz (ETSI EN 301 893 v2.1.1 (2017-05)), and (3) 1000 mW (30 dBm) for (5470–5725) MHz (ETSI EN 301 893 v2.1.1 (2017-05)) [18,19].

Bluetooth is a technology for short-range wireless communication between various electronic devices. It is an open standard described in the IEEE 802.15.1 specification that includes three transmission power classes with a range of 100 (class 1), 10 (class 2) and 1 (class 3) metre in open space. According to ETSI EN 300 328 V2.2.2 (2019-07), the limits of EIRP from Bluetooth devices are 100 mW (20 dBm), 2.5 mW (4 dBm) and 1 mW (0 dBm) for class 1, class 2 and class 3 devices, respectively. The standard uses the same ISM frequency band as Wi-Fi 2G. For IoT applications, e.g., medical, security-related, remote meter reading and position determination, Bluetooth Low Energy (LE) technology has been developed, in which the power consumption and data transmission speed have been significantly reduced, while increasing the range up to 400 m indoors (1000 m outdoors).

### 1.4. The Metrics of Thermal Effects of EMF Exposure and Evaluation

It is well known that exposure to a high intensity electromagnetic field (EMF) produces biological effects in human tissues, potentially leading to adverse health consequences [20,21,22,23,24]. There are multiple factors to consider with respect to the biophysical effects of the influence of EMF, as well as their significance with respect to the safety and health of the population. The behaviour of human tissues varies with the frequency of the EMF and bioelectromagnetic interactions may be thermal from tissue being heated by absorbed electromagnetic energy, or more complex non-thermal [21,23,25,26,27,28,29]. The most commonly-accepted measure of direct biophysical thermal effects is the specific energy absorption rate (SAR, in watts per kilogram, W/kg—body-averaged or local SAR values). The relevant SAR limits for the protection against electromagnetic hazards to the general public or workers (at a level five times higher) are considered in the evaluation of exposure to EMF with respect to the minimum safety requirements [22,23,24]. General public limits of time averaged SAR are 0.08 W/kg—body-averaged SAR (WBSAR); 2 W/kg—local head and torso SAR and 4 W/kg—local limb SAR, both averaged over 10 g cubic mass (SAR10g). They are applicable for EMF exposure at a frequency from 100 kHz up to 6 GHz (International Commission on Non-Ionizing Radiation Protection (ICNIRP) 2020, IEEE C95.1-2019) or up to 10 GHz (ICNIRP 1998) [22,23,24]. In the reported studies, the SAR averaged over the whole head (WHSAR) was determined due to the limitation of the human body model to the head model only. It should be noted that ICNIRP 1998 limits are still the most important because they were referenced in various binding legislative documents, such as for example European Directive 2013/35/EU [30].

Compliance with SAR limits may be evaluated by compliance with the exposure limits: incident electric field strength (E, in V/m) and incident magnetic field strength (H, in A/m), or alternatively incident power density (S_inc_, in (W/m^2^) [22,24]. The newest guidelines suggest using E or H for frequencies up to 30 MHz, E and H or S_inc_ for frequencies from 30 MHz up to 2 GHz, and using S_inc_ for higher frequencies [23].

Wearable IoT devices are often used in close proximity to the human body; in such cases, in accordance with international safety guidelines, a SAR compliance assessment is required, even when the incidental EMF has already been evaluated [23,24]. The application of the aforementioned higher SAR limits of workers’ EMF exposure also triggers the application of specific EMF safety programs including various safety measures required by labour law for workers exposed to strong EMF (such as EMF exposure evaluation at a particular workplace and its limitation, safety training of exposed workers, etc.) [30]. Because workers using wearable IoT devices are usually not covered by such EMF safety programs in the work environment and would not be suitably trained to mitigate hazards from EMF exposure, it is appropriate to carry out SAR compliance assessments against general public limits, when it is expected that only weak EMF exposure is found.

### 1.5. The Aim

The aim of this study was to evaluate the absorption in the user’s head of an EMF emitted by a wearable IoT device using Wi-Fi and/or Bluetooth communication technologies—i.e., small electronic devices emitting EMF of up to 100 mW—in order to test the hypothesis that EMF emitted by these devices have an insignificant influence on humans, and to compare the levels of such EMF absorption in various scenarios when using these device.

## 2. Materials and Methods

### 2.1. Numerical Model of EMF Source and Its Validation

**Figure 3:**
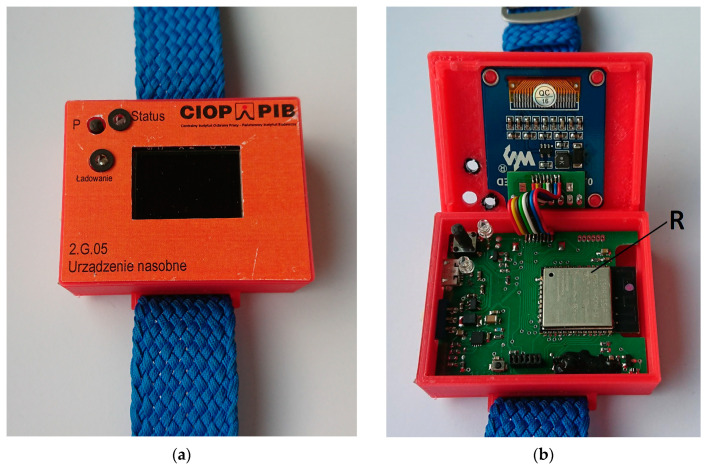

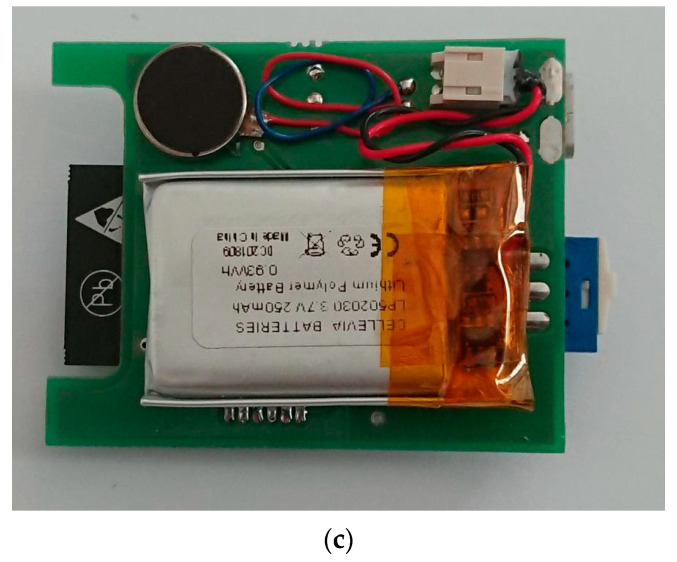
(**a**) Wearable device; (**b**) its internal construction top (R—radiofrequency module, ESP32-WROOM-32); (**c**) bottom view.

The considered WSNs (using sample devices developed at CIOP-PIB) consist of a specific type of wearable device that is able to perform Wi-Fi and/or Bluetooth connections [15,16,17]. Wearable devices are worn by workers and used to alert them to any hazards, or to inform them of the necessary safety measures in the monitored area, for example to alert workers using hearing protectors against approaching vehicles. The electronic circuits of both wearable devices consist of one double-sided printed circuit board (PCB). A radiofrequency module is mounted on the PCB’s top side, as shown in Figure 3. The PCB has a cut out in the antenna area of the radio module. A small Li–Po cell, a vibration motor and a miniature loudspeaker are located below the PCB. The case for both devices was made of polylactic acid (PLA) polymer on a 3D printer and has walls 1.8 mm thick.

The heart of both wearable devices in the considered applications is the commercial radiofrequency (RF) module ESP32-WROOM-32. This module was chosen because it enables wireless communication in both Wi-Fi and Bluetooth standards (necessary in considered WSNs to transmit data to the main network unit or receive data to alert workers to hazards and to localise workers) and is easily programmable and available. Moreover, it is based on system-on-chip (SoC), with relatively high computing power equipped with flash memory. This allows a wearable device to be built based on the radio module itself, without the need for an additional microcontroller.

**Figure 4:**
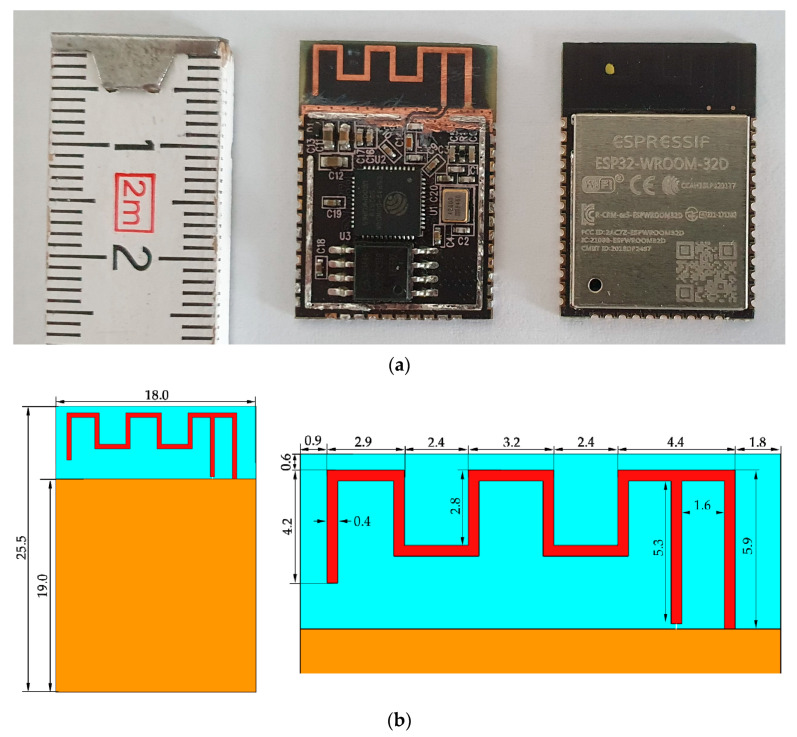
(**a**) View of physical wireless communication module (ESP32-WROOM-32 radiofrequency (RF) module) and (**b**) dimensions (in millimetres) of developed numerical model of meandered inverted-F antenna (MIFA) (**right**) and dimensions of substrate and ground (**left**) of RF module used in Internet of Things (IoT) wearable devices.

The ESP32-WROOM-32 is equipped with an omnidirectional meandered inverted-F antenna (MIFA). It should be mentioned that other RF modules from the same manufacturer, such as the ESP32-S2, are also equipped with the same MIFA. Therefore, the investigation results presented in this article also apply to such modules or modules of a similar structure. The MIFA was designed to operate in the ISM frequency band (2400–2483.5 MHz) and supports Wi-Fi 2G and Bluetooth communication technologies compliant with the requirements of international standards IEEE 802.11 and IEEE 802.15.1. Maximum EIRP declared by the manufacturer for the ESP32-WROOM-32 module is 100 mW (20 dBm) for Wi-Fi communication (i.e., the maximum value for which use does not require special administrative permission) while for Bluetooth it is 8 mW (9 dBm) (i.e., the value lower than the maximum one not requiring special administrative permission) [18]. The numerical model of the MIFA was an antenna with outer dimensions of 15.3 × 5.9 mm and ground of 18.0 × 19.0 mm placed on a substrate of 18.0 × 25.5 mm (Figure 4). Copper with a thickness of 0.035 mm, an electric conductivity of 5.813 × 10^7^ S/m and a relative permittivity of 1 was used for the antenna and ground; FR-4 (glass-reinforced epoxy laminate material) with a thickness of 0.7 mm, an electric conductivity of 3.8 × 10^−3^ S/m and a relative permittivity of 4 was used as the substrate.

Pilot tests were carried out for exposure scenarios with the numerical model of the wearable device with the MIFA plane at a distance of 7 mm from the model of the head. This consisted of the RF module and a battery placed below it, as shown in Figure 3c (i.e., the battery located between the numerical model of head and the RF module in such a way that it did not cover the MIFA) and with wearable devices consisting only of an RF module. The results showed insignificant differences between the obtained maximum SAR10g values (0.2%) and the WHSAR (SAR evaluated as averaged over the entire exposed head) values (1%). The results obtained from the pilot simulations supported the use of a simplified structure of the model of the wearable device (only the model of the MIFA without a battery), as in other published studies [31,32]. On the basis of these results for further investigations, the electronic elements, housing, fixtures and battery were not included in the numerical model of the considered wearable device. It was assumed that their role may be neglected in simulations of SAR values in the head of a user of a wearable device.

**Figure 5:**
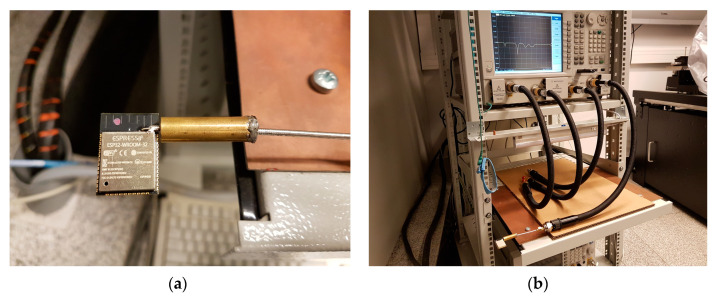
The laboratory measurements of the S11 parameter: (**a**) the sleeve balun choke used to suppress unwanted surface currents propagating onto the outer shield of the feeding cable and (**b**) equipment used during the S11 measurements.

The numerical model of the described MIFA was validated by laboratory tests of the undisturbed (in free space) S11 reflection coefficient parameter. The reflection coefficient of the antenna being tested was measured using an Agilent PNA-X N5242A vector network analyser (Figure 5). During the measurements, the ESP32-WROOM-32 module was placed a sufficient distance away from other objects to obtain undisturbed results. Additionally, time domain gating was applied to eliminate the influence of unwanted reflections (e.g., from the SubMiniature version A (SMA) connector). This additional data processing was possible because the S11 was measured in a wide frequency range from 100 MHz to 20.1 GHz (with 1 MHz spacing). The uncertainty of the S11 measurement in a given frequency band was estimated using the software provided by the vector network analyser manufacturer, as not exceeding ±2.5% (K = 1) for data expressed in a linear scale.

### 2.2. Human Body Numerical Model

Due to the considered exposure scenarios in which the MIFA is located close to the head, the study was limited only to the numerical model of a head. The head was modelled as a multi-layer ellipsoid with dimensions corresponding to the dimensions of the 50th percentile male head of the Polish population: along the long axis—238 mm, along the transverse axis—159 mm and along the sagittal axis—190 mm [33,34,35]. The model includes layers with density and dielectric parameters at a 2.45 GHz frequency adopted from the Foundation for Research on Information Technologies in Society (IT’IS, Zurich, Switzerland) Database of Tissue Properties corresponding to the skin, subcutaneous adipose tissue (SAT)/fat tissue, bone (skull bones) and the internal part corresponding to brain grey matter (Table 1) [36]. The thicknesses of particular tissue layers were the median values for people: 4 mm for the skin, 2 mm for the SAT/fat, 9 mm for the skull bone and inner ellipsoid volume corresponding to brain [34].

### 2.3. Exposure Scenarios

The considered wearable devices may be worn by a worker in a way comparable to a watch or some kind of band, or built-in or attached to various items of work clothes or personal protective equipment such as a helmet. In the same applications, it is necessary to use two cooperating wearable devices simultaneously, i.e., located on the wrist and at the head (for example, as a headband or at helmet) [17]. Among these locations, the worst-case of exposure to EMF emitted by the RF module of wearables is related to their location at the head (the limits of SAR10g for head exposure are half that of the limits for limb exposure, 2 W/kg vs. 4 W/kg for the general public), and further research was focused on this.

**Figure 6:**
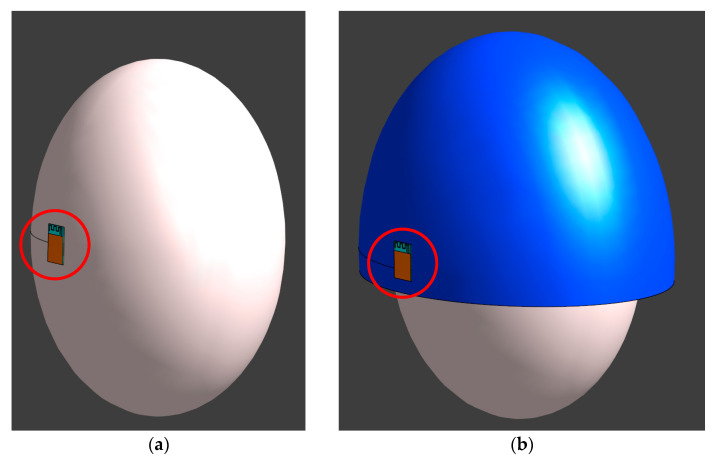
Exposure scenarios with the RF module located (**a**) on the headband; (**b**) attached to a helmet (the RF module is circled).

The investigations covered exposure scenarios with the MIFA located at the side of the head: (1) on the headband with the antenna plane at a distance of 2 mm from the head, or (2) attached to a helmet of the type required, for example, in an industrial environment, with an antenna plane at a distance of 20 mm from the head (Figure 6). High-density polyethylene (HDPE) with a thickness of 2 mm, an electric conductivity of 5.0 × 10^−4^ S/m and a relative permittivity of 2.25 was used for the helmet.

### 2.4. Numerical Simulations

To quantify the EMF absorption, the specific energy absorption rate (SAR) values were calculated in a multi-layer ellipsoidal model of the human head (involving skin, fat, skull bone and brain layers).

The simulations were carried out by Sim4Life software (Zurich Med Tech, Zurich, Switzerland) using finite-difference time-domain (FDTD) solvers and the finest resolution of 0.005 mm was set for the antenna, and 1 mm was set for the numerical model of the head and other elements of exposure scenarios. The resolution of the numerical model of the head was finer than the minimum resolution established for the evaluation of SAR required by the International Electrotechnical Commission (IEC) standard 62232-2011—the resolution of the human body model was better than 1/15 of the wavelength in tissues [37]. The uncertainty of the numerical simulations was estimated as not exceeding ±(20–25)% (K = 1) and was in the range compliant with state-of-the-art in SAR numerical simulations [37,38,39].

The SAR values were calculated in the numerical model of a head exposed to RF EMF emitted by the ESP32-WROOM-32 RF module, averaged over the whole head or averaged over any 10 g of continuous tissue (localised) [22,23,30,38]. The averaging algorithm according to IEC/IEEE 62704-1:2017 was used to obtain localised SAR values [38]. This algorithm is appropriate for use in a compliance assessment with the SAR limits established by IEEE C95.1 and ICNIRP 1998 [22,24].

## 3. Results

**Figure 7:**
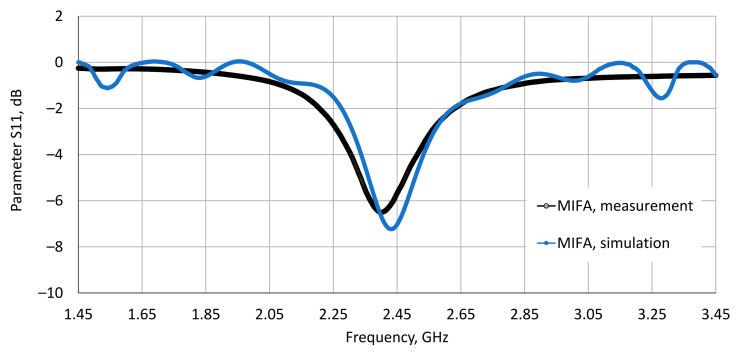
The S11 parameter, measured from the wireless communication module (the ESP32-WROOM-32 RF module) in the laboratory and determined using numerical simulations of the developed numerical model of the MIFA, used in the considered IoT wearable devices.

The numerical model of the described MIFA was validated by laboratory tests of the undisturbed (in free space) reflection coefficient S11 parameter. The results of an experimental validation showed sufficient agreement of the obtained centre (resonance) frequency (RF module matching frequency), with a difference of 1.3% between the measurement and simulations (2.400 GHz—measurements vs. 2.432 GHz—simulations) (Figure 7).

**Figure 8:**
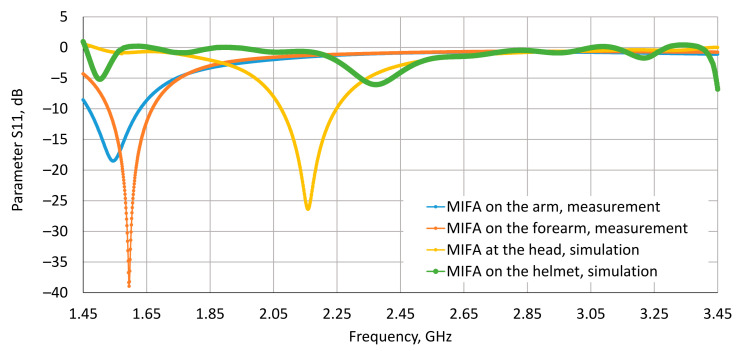
The S11 parameter, measured in the laboratory and determined using numerical simulations of the developed numerical model of the wireless communication module (RF module) used in IoT wearable devices located near various parts of the worker’s body.

In the cases with the MIFA close to the body (on the arm, forearm and at the head), it was experimentally observed that the dielectric parameters of the tissues had a significant influence on the S11 parameter (Figure 8). The resonance frequency of the considered antenna and the antenna performance varied significantly near the surface of the body. Our study showed that resonance frequency varied among 1.545 GHz, 1.595 GHz and 2.159 GHz for the MIFA located on the arm, forearm and at the head respectively. The reason for this is that the human body acts as a new layer of substrate with a complex structure and surface, as has also been observed in other studies [31,40]. The results obtained show that locating the MIFA at a very short distance (a few millimetres) from the human body causes a high mismatch with the desired operating frequency and the antenna’s performance.

Table 2 shows the results of the numerical simulations of SAR values related to EMF exposure near the ESP32-WROOM-32 RF module evaluated at an input power to the MIFA of 100 mW at a frequency of 2.45 GHz (analysed with respect to continuous exposure), i.e., evaluated at the worst-case exposure scenario with respect to the level of EMF emission from the considered RF module and the rules of evaluating thermal load from human exposure to EMF.

The obtained results show SAR values in the case of the RF module located in the headband up to 10 times higher in comparison to its location on the helmet. Furthermore, in this exposure case, for an input power to the antenna over 50 mW, SAR10g values may exceed the limits for the general public (2 W/kg), while for an input power to the antenna over 250 mW, SAR10g values may also exceed the limits for occupational exposure (10 W/kg). It was observed in the results of numerical simulations that antenna performance drops dramatically when it is located very close to tissues (only 6.1 mW EIRP @ 100 mW of input power to antenna, compared to 350 mW when used on the helmet).

## 4. Discussion

The measurements of antennas in portable radios are often challenging. Due to the small size (with respect to the wavelength) of the antenna being tested and the finite ground plane of the PCB, the impedance measurements suffer from the influence of the attached RF cable [41]. Therefore, a sleeve balun with a diameter of 8 mm placed on the RG405 coaxial cable close to the antenna was used (Figure 5). This suppresses unwanted surface currents propagating onto the outer shield of the feeding cable. The length of the sleeve balun choke was 0.23 λ, which is the optimal length to achieve maximum insulation [42].

According to ETSI EN 300 328 V2.1.1:2017-058 for transmission systems operating in the 2.4 GHz ISM band, the limit of EIRP is equal to 100 mW (Wi-Fi 2G, Bluetooth class 1) [18]. In the case of an RF module continuously using the maximum EIRP, located on headband, the values of SAR10g may be 32 times and 6 times higher than the limits for the general public (2 W/kg) and occupational exposure (10 W/kg), respectively. In fact, these values are unlikely to occur due to the technically limited input power to the analysed MIFA (built in the ESP32-WROOM-32 RF module).

The analysed frequency band (2.4 GHz—ISM) is very close to the frequency bands used by LTE: 2100 (1920–1980 MHz uplink; 2110–2170 MHz downlink), 2300 (2305–2315 MHz uplink; 2350–2360 downlink) and 2600 (2500–2570 MHz uplink; 2620–2690 MHz downlink) MHz. The limit of unlicensed use of EIRP is the highest for LTE devices and equal to 200 mW (ETSI TS 136 101 V15.9.0 (2020–02) [43]. Consequently, the obtained results of SAR values near the modelled MIFA at 2.45 GHz may also be used to roughly estimate the SAR values for the use of similar RF transmitters in these LTE frequency bands, which may have an EIRP up to twice as high as it is allowed following the technical specifications for Wi-Fi 2G and Bluetooth devices. Consequently the worst-case SAR values caused by the use of MIFA in the LTE system are also twice as high as in analysed Wi-Fi/Bluetooth systems. The most realistic time-pattern of EMF emission in analysed WSN application is not continuous, but the worst-case exposure level as discussed in this section also needs attention. Continuous emission from MIFA is allowed in the software controlling the considered RF module; it may be used intentionally, e.g., when communication within the WSN is tested, but also unintentionally because of programming errors or technical malfunctions of the system.

Only wearable IoT devices (similar in construction to the investigated device) using Wi-Fi/Bluetooth communications technologies emitting at below 3 mW EIRP may be considered as environmentally insignificant EMF sources (i.e., their direct biophysical influence on the body of their user seems to be weaker than the limits of SAR caused by EMF influence provided by international safety guidelines). When higher EIRP is required for sufficient use of particular applications of wearable IoT devices (e.g., when WSN is improving safety in the work environment), the workers using such wearables need to be counted as EMF-exposed workers, together with application of an EMF safety program, as labour law defines.

## 5. Conclusions

The results of this work show that the absorption in the user’s head of an EMF emitted by a wearable IoT device using a MIFA, for example applied to alert workers against hazards in the work environment, may have a significant influence on humans when used directly on the head [44]. Locating the MIFA on a helmet may sufficiently reduce the level of the IoT device user’s exposure to EMF. Based only on the presented results, it is impossible to indicate the best (safe in terms of lowest SAR values) exposure scenario. This requires further research, taking into account more detailed exposure scenarios such as anatomically-based models of the human body, other locations for wearable devices on the worker (e.g., on wrist, chest, arm, leg, etc.), various antenna plane locations against the body surface (parallel or normal) and device ground-plane normal or parallel to the skin, rather than investigating more sophisticated models of wearable IoT devices.

These studies indicate that locating the wearable device at a very short distance (a few millimetres) from the human body causes a high mismatch with the desired operating frequency and the antenna’s performance. This may result in the need to increase the input power to the device antenna to ensure a stable radio-link between the device and other WSN components, the access point or the main unit. A consequence of this is also an increase in the SAR value. Hence, a good and safe wearable IoT device is one that has been designed and optimised for the dedicated use of the device (with low power needed to set a stable radio-link receiver-transmitter), for example in a headband or as a watch. For this reason, research on new technical solutions (new antenna designs, new materials and designs of IoT devices) is recommended to improve the parameters of radio links created with the use of the IoT device (by means of its operation at low EMF emission, which also means automatically reducing the SAR value in the user’s body).

## Figures and Tables

**Table 1:** Dimensions and parameters at 2.45 Hz of tissues considered in the numerical model of the head (a multi-layer ellipsoid).

Tissue	Dimensions, ^(1)^ mm	Layer Thickness, mm	Density, kg/m^3^	Electric Conductivity, S/m	Relative Permittivity
skin	238 × 159 × 190	4	1209	1.464	38.0
fat	230 × 151 × 182	2	911	0.268	10.8
bone	226 × 147 × 178	9	1908	0.394	11.4
brain	208 × 129 × 160	inner volume	1045	1.808	48.9

^(1)^ Dimensions—outer dimensions of particular layers in numerical model of head expressed as dimensions along long axis × transverse axis × sagittal axis, in mm.

**Table 2:** Specific energy absorption rate (SAR) values in the head of the user of an ESP32-WROOM-32 RF module equipped with a MIFA, for an input power to the antenna of 100 mW @ 2.45 GHz.

Exposure Scenario	WHSAR, ^(1)^ W/kg	SAR10g, ^(2)^ W/kg	EIRP, ^(3)^ mW
RF module on the headband	0.035	4.0	6.1
RF module on the helmet	0.005	0.4	350

^(1)^ WHSAR—SAR evaluated as averaged over the entire exposed head; ^(2)^ SAR10g—maximum local SAR averaged over 10g of mass of any continuous tissue; ^(3)^ EIRP—equivalent isotropically radiated power.
